# Gastrointestinal nematodes of dairy goats, anthelmintic resistance and practices of parasite control in Northern Italy

**DOI:** 10.1186/1746-6148-10-114

**Published:** 2014-05-19

**Authors:** Sergio Aurelio Zanzani, Alessia Libera Gazzonis, Annarita Di Cerbo, Marian Varady, Maria Teresa Manfredi

**Affiliations:** 1 Department of Veterinary Science and Public Health, Università degli Studi di Milano, Milan, Italy; 2 Parasitological Institute of the Slovak Academy of Science, Košice, Slovak Republic

**Keywords:** Parasites, Gastrointestinal nematodes, Anthelmintic resistance, Goat, Italy

## Abstract

**Background:**

Gastrointestinal nematodes (GINs) are one of the main constraints to ruminant production worldwide. Anthelmintic resistance (AR) has been reported in goats throughout Europe, yet little is known about the AR status in Italy. The aims of the study were: i) determine the frequency of AR in GINs in goat flocks in Northern Italy, Italy, ii) survey goat farmers on the current practices of parasite control, iii) update the species composition of the gastrointestinal helminthofauna. Thirty three flocks were enrolled and 1288 individual fecal samples were collected. Based on the egg per gram (EPG), 15 flocks were selected to evaluate the presence of AR in GINs with the Fecal Egg Count Reduction Test (FECRT). A questionnaire surveyed 110 dairy goat farmers to acquire information about farm management and drenching practices against GINs. Further, the gastrointestinal tracts of 42 goats were analyzed.

**Results:**

The FECRs indicated that five of the 15 flocks had problems of AR, which was identified in all two of the anthelmintic classes tested. Resistance and suspected resistance was found in 40% of the flocks selected for AR testing that were treated with benzimidazoles while 20% of the flocks treated with eprinomectin had resistant GINs. *Teladorsagia/Trichostrongylus* L3 were isolated from the post-treatment coprocultures of all flocks with resistance but not from the flock with suspected oxfendazole resistance. Treatments against helminths were performed once annually in 73.63% of the flocks, but 20.00% of farmers declared not regularly treating their goats every year. Annual treatments usually occurred in autumn or winter at dose rate for sheep. *Te. circumcincta*, *H. contortus*, *Tr. colubriformis*, *Skrjabinema caprae* and *Oesophagostomum venulosum* were the most abundant and prevalent species of the gastrointestinal tract.

**Conclusions:**

Strategies to prevent the development of AR should be widely adopted in Northern Italy. Further, farmers and practitioners should be educated about the importance of using the correct dose rates in goats. In addition, the presence of highly pathogenic GINs coupled with high worm burden in all sector of gastrointestinal tract and the prevalence values further suggest that improved diagnosis and active surveillance of GINs infection is needed.

## Background

Gastrointestinal nematodes (GINs) remain one of the main constraints to ruminant production, since they can causing reduction in skeletal growth, live-weight gain and in milk yield [1,2]. GINs infection is of economic importance also in goat farming worldwide and the peculiarities of goat-nematode interactions could affect the control strategies of GINs. In particular, goats can be significantly more heavily infected than sheep and both the acquisition and expression of immune responses against GINs are less efficient in goats than sheep [3]. Further, the control of GINs infection in goats is still largely based on use of drugs at regular intervals and currently is made more complicated by the presence of resistant nematodes to one or more type of drugs. The phenomenon of anthelmintic resistance (AR) is spread in many countries with differences in prevalence and some data showed that GINs develop anthelmintic resistance more rapidly in goats [4,5]. A few recent studies have examined the gastrointestinal parasites of goats [6,7], but none have described the control practices used on dairy-goat farms in Northern Italy and especially in Lombardy. Anthelmintic resistance (AR) against benzimidazoles has previously been reported in this area but is considered rare [8]. Knowledge of the presence of this phenomenon and of the control practices that are normally employed by farmers could be useful for improving both the management of gastrointestinal parasites and the milk production of grazing goats in marginal or mountain areas. Nevertheless, knowledge of the specific composition of the gastrointestinal helminthofauna can also supply valuable information for parasite control. The main aim of this study was to evaluate the presence of AR in the gastrointestinal nematodes of various breeds of dairy goats in Northern Italy by the fecal egg count reduction test (FECRT) and to determine which species could be implicated in resistance using larval cultures. A secondary aim was to survey goat farmers on the current practices of parasite control on farms in Northern Italy. Another goal was to update the species composition of the gastrointestinal helminthofauna by identifying the adult nematodes recovered from gastrointestinal tracts.

## Methods

A survey was performed with a questionnaire from November 2010 to June 2011 in Lombardy, a region of Northern Italy (45°40’N, 9°30’E). Members of SATA, a regional association offering zootechnical and veterinarian support to breeders, were involved in the study. The majority of surveyed farms were in the areas of Northern Italy most suitable for goat breeding.

All animal procedures used in this study were approved by the Milan University Institutional Animal Care and Use Committee.

### Evaluation of AR

#### Fecal egg count reduction test (FECRT)

Individual fecal samples were collected from the rectums of 1288 dairy goats from 33 flocks. All animals were older than two years of age. The parasitological analyses used a modified McMaster method [9,10], with 40.5 ml of a sodium nitrate and sucrose-based flotation solution (specific gravity = 1.30) and 4.5 g of feces. The numbers of eggs per gram (EPG) were calculated. Based on the EPGs, 15 flocks were selected to evaluate in vivo the presence of AR in GINs with the FECRT, in accordance with the guidelines of the World Association for the Advancement of Veterinary Parasitology (WAAVP) [11,12].

Benzimidazoles and probenzimidazoles (BZs) and macrocyclic lactones (MLs) were the classes of anthelmintic selected for evaluation in this survey based on the information obtained from the questionnaire. Albendazole (ABZ) (7.5 mg/kg body weight (bw)), oxfendazole (OFZ) (10 mg/kg bw), febantel (10 mg/kg bw) and netobimin (15 mg/kg bw) were tested in ten flocks. Ivermectin (IVM) and moxidectin (MOX) at 0.4 mg/kg were administrated to goats of one and two flocks, respectively. Finally, eprinomectin (EPM) at an extra-label dose of 1 mg/kg bw was tested in two flocks. All drugs were orally dosed except EPM, which was applied with a pour-on formulation. The dose rates of the anthelmintics were twice as high as those for sheep or cattle. The body weight of the visually heaviest goat of each flock was estimated to determine the anthelmintic dose rate. The number of dosed animals in each flock, based on the recommendations provided by Coles et al. [11], varied from 20 to 32 goats depending on flock size.

Individual fecal samples were collected on the day of treatment (T1). T1 mean EPG and standard deviation were calculated for each flock. FECRTs were performed only on goats with EPGs ≥ 150 at T1. Individual post-treatment fecal samples (T2) were collected after 8–10 days from goats treated with BZs and after 14–17 days from goats treated with MLs. The fecal egg count reduction (FECR) was calculated with the formula proposed by Cabaret and Berrag [13], in which each host serves as its own control:

$$\text{FECR}=\left(1/n\right)\Sigma \left(100x\left(1-\left[T_{i2}-T_{i1}\right]\right)\right)$$

where *T*_*i2*_ is post-treatment and *T*_*i1*_ is pre-treatment EPG in host *i* from a total of *n* hosts. The tested population of nematodes was classified as resistant when the FECR was <95% and the lower limit of the 95% confidence interval (CI) was <90% or as “suspected resistant” when the FECR was <95% or the lower limit of the 95% CI was <90% [11].

#### Larval cultures

Pre- and post-treatment larval cultures were performed from pooled fecal samples for each flock; pools were composed with feces collected from each animal of the group. The feces sampled at T1 and T2 were incubated for 10 days at 25°C in a large vessel, ensuring adequate moisture (50-80%), and third-stage larvae (L3) were recovered from the coprocultures by a Baermann technique [9]. The first 100 randomly selected L3 of each pool were then examined with a light microscope (Axioscope 2, Zeiss) and were identified to the generic level as *Teladorsagia/Trichostrongylus*, *Oesophagostomum/Chabertia*, *Haemonchus*, *Nematodirus*, *Bunostomum* and *Cooperia*, according to MAFF [9] and van Wyk et al. [14]. When fewer than 100 L3 were isolated from a pool, the percentage of larval type was calculated on the basis of the counted L3.

#### Farm questionnaire

A questionnaire surveyed 110 dairy goat farmers from 51, 43, 10 and six farms from eastern (Bergamo (BG) and Brescia (BS) provinces), western (Como (CO), Lecco (LC) and Varese (VA) provinces), northern (Sondrio (SO) province) and southern (Pavia (PV) province) areas of Lombardy, respectively. Each farmer was asked about their farm management, including the presence and dimensions of pastures, demography and breed of goats, the presence of other species on the farm (e.g. cattle) and drenching practices against GINs, such as treatment times, frequency, products and dosages of anthelmintic drugs. Farm veterinarians contributed to answering these last questions.

#### Composition of gastrointestinal helminthofaunas

The gastrointestinal tracts of two or three goats from the 15 flocks previously selected to evaluate the presence of AR were collected at abattoirs. The species composition and abundance of abomasal and intestinal nematodes of 42 goats were analyzed. Abomasa and small and large intestines were examined separately; they were opened and the contents were filtered through metallic sieves to collect adult helminths [9]. Ten percent of the content was examined with a stereomicroscope, and the extracted worms were counted. Cleared male worms were identified according to established descriptions [15-20]. The nomenclature used for Ostertagiinae was that proposed by Drózdz [15] and Durette-Desset [21]. The numbers of nematodes in the content fractions were adjusted to the total volumes from the abomasa or intestines. The total number of nematodes (males and females) and the number of individuals of each species based on the taxonomy of adult male worms were recorded. Epidemiological indexes, mean abundance (A) and prevalence (P), were calculated for each species of helminth when possible [22]. The helminth community structure was examined by the importance value, I, that was calculated for each of the helminth species according to Thul et al. [23].

## Results

### Evaluation of AR

Adult goats of the 15 flocks selected for testing AR had a mean pre-treatment EPG of 426.93 (±249.11) (Table 1). Baermannized L3 isolated from pre-treatment pools of individual fecal samples showed that *Teladorsagia/Trichostrongylus* were the most common GINs in the study area: *Teladorsagia/Trichostrongylus* L3 were recovered from 13 of the 15 flocks (86.6%) and were the most prevalent GINs in eight of the 13 flocks (61.5%) infected with these worms*. Oesophagostomum/Chabertia* and *Haemonchus* L3 were recovered from 11 (73.3%) and nine (60%) flocks, respectively, and *Haemonchus* was the most prevalent GIN in six of the nine flocks (66.6%) infected with this species. *Bunostomum*, isolated from five flocks (33.3%), was the most prevalent GIN in flock 11. According to Table 1*Nematodirus* L3 were isolated only from flock 3, and *Cooperia* L3 were not found in any flock.

**Table 1:** Mean eggs per gram (EPG) from fecal egg counts (FEC) and third-stage larvae identified in the goats of flocks selected for testing for anthelmintic resistance

**Flock no.**	Province*	**Area**	**Pre-treatment FEC**		**Pre-treatment coproculture**				
			**Mean EPG**	**±SD°**	***Telad/Trich***	***Oesop/Chab***	***Haem***	***Nemat***	***Bunost***
1	BS	E	248	100	96	4	0	0	0
2	BS	E	1317	1040	0	3	97	0	0
3	BS	E	250	62	81	0	0	9	10
4	BS	E	424	294	82	5	13	0	0
5	BG	E	307	397	78	17	5	0	0
6	BG	E	720	484	30	27	43	0	0
7	BG	E	261	110	81	19	0	0	0
8	BG	E	367	245	84	0	0	0	16
9	BG	E	468	264	32	29	39	0	0
10	BG	E	289	225	92	4	0	0	4
11	VA	W	312	144	29	0	0	0	71
12	VA	W	494	350	0	0	100	0	0
13	VA	W	400	397	24	7	69	0	0
14	VA	W	218	62	20	4	76	0	0
15	SO	N	329	85	77	5	12	0	6

*BS = Brescia, *BG* = Bergamo, *VA* = Varese, *SO* = Sondrio, ° = standard deviation, *Telad/Trich = Teladorsagia/Trichostrongylus, Oesop/Chab = Oesophagostomum/Chabertia, Haem = Haemonchus, Nemat = Nematodirus, Bunost = Bunostomum*.

The percentages of the FECRs and the 95% confidence intervals of the fifteen investigated flocks are shown in Table 2. The FECRs after treatment indicated that 4 cases and 1 doubt of the 15 flocks (33.33%) had problems of AR, which was identified in the tested anthelmintic classes. One of the three flocks dewormed with ABZ had a post-treatment FECR fitting the criteria for resistance proposed by Coles [11]: the FECR was 81.11% and the lower 95% CI limit was 69%. One of the two flocks dewormed with OFZ was classified as “suspected resistant” (FECR = 95.17, lower 95% CI limit = 88%). The FECR was 100% in all flocks treated with netobimin, demonstrating the complete efficacy of this drug. Both flocks treated with febantel had resistant worms, with very low FECRs (40.52 and 76.01%, respectively) and lower 95% CI limits (58% and −3%, respectively). ML treatments were effective in four of five flocks; one of the two flocks treated with EPM was classified as resistant, having a FECR of 87.43% and a lower 95% CI limit of 76%. All areas of Northern Italy had flocks with FECRs less than 100%. The flocks with resistance or suspected resistance were all located in the eastern provinces. *Teladorsagia/Trichostrongylus* L3 were isolated from the post-treatment coprocultures of all flocks with resistance but not from the flock with “suspected” OFZ resistance. A low percentage of *Haemonchus* larvae (25%) were isolated from the coproculture of the flock with EPM resistance (Table 2).

**Table 2:** Fecal egg count reduction (FECR), 95% confidence intervals (CI) and third-stage larvae (L3) identified in post-treatment coprocultures in selected goat flocks

**Anthelmintic class**	**Drug**	**Flock no.**	**FECR (%)**	**95% CI**	**Status**	**Post-treatment coproculture**	
						**L3 type (%)**	**No. L3 seen**
BZs	ABZ	1	100	°	S		0
	OFZ	3	100	°	S		0
	OFZ	4	95.17	88-100	SR**		0
	ABZ	5	81.11	69 -94	R*	*Teladorsagia/Trichostrongylus* (100)	23
	ABZ	6	100	°	S		0
	Febantel	7	76.01	58-94	R*	*Teladorsagia/Trichostrongylus* (100)	15
	Febantel	8	40.52	−3 to 84	R*	*Teladorsagia/Trichostrongylus* (100)	38
	Netobimin	11	100	°	S		0
	Netobimin	12	100	°	S		0
	Netobimin	13	100	°	S		0
MLs	IVM	10	100	°	S		0
	MOX	14	98.82	97-100	S		0
	MOX	15	98.57	96-100	S		0
	EPM	2	99.32	98-100	S		0
	EPM	9	87.43	76-99	R*	*Teladorsagia/Trichostrongylus* (75), *Haemonchus* (25)	16

*BZs*, Benzimidazoles and probenzimidazoles; *MLs*, Macrocyclic lactones.

*ABZ*, Albendazole; *OFZ*, Oxfendazole; *IVM*, Ivermectin; *MOX*, Moxidectin; *EPM*, Eprinomectin.

S, susceptible.

*resistant (R) flock: FECR <95% AND lower 95% CI limit <90%.

**suspected resistant (SR) flock: FECR <95% OR lower 95% CI limit <90%.

°95% CI not calculated because the reduction was 100%.

### Farm questionnaire

The questionnaire survey indicated that 42.73% (47/110) of the goat farms practiced grazing, and the majority of farms with pastures (60.5%) were in the western area. Pastures had a mean area of 11.63 ha (range, 1–50 ha) and altitudes from 200 to 1500 m a.s.l. Goats usually grazed from March or May to October or November during the day (or at night in the hottest months) and were kept in the fold at night (or day in the hottest months), depending on the region. Intensive breeding was the most common system in farms from southern (100%) or eastern (90.2%) areas, whereas only 55.8% or 50% of farms from western or northern areas, respectively, had this type of farming. Farms in the eastern and northern areas had the largest mean flock sizes (84.65 and 100.85, respectively); these farms also had the highest number of does (Table 3). Only one breed of goat was farmed in 89.10% (98/110) of the flocks. Eleven of 110 (10%) farms had does belonging to autochthonous breeds (Orobica, Nera di Verzasca, Bionda dell’Adamello and Frisa Valtellinese), but only five farms raised only one these breeds. The autochthonous breeds were raised on farms of northern (3/11), eastern (5/11) and western (3/11) Lombardy according to the geographical area from which they originated. A large number of farmers (49.10%) confirmed maintaining other species (cattle or sheep) on their farms, but generally in low numbers. Goat farms were mainly specialized for cheese production. The percentage of farms producing milk for cheese varied from 70% in northern areas to 100% in southern areas (Table 3).

**Table 3:** Characteristics of goat farms and flock demographics

**Location of farm**	**No. farms surveyed**	**Breeding system (% of farms)**			**Pasture (% of farms)**	**Mean flock size**	** Main breeds**	**Production of cheese (% of farms)**
		E*	**SE°**	**I**^**§**^		**(Does, range)**		
Eastern area	51	5.8	1.9	90.2	31.4	84.65 (18–840)	Alpine	82.35
							Saanen	
							Orobica	
Northern area	10	0	30	50	40	100.85 (21–190)	Alpine	70
							Frisa Valtellinese	
Western area	43	34.8	9.3	55.8	60.5	40.97 (4–156)	Alpine	86
							Saanen	
							Nera di Verzasca	
Southern area	6	0	0	100	16.6	39.20 (7–93)	Alpine	100

* = extensive, goats are free to graze and browse all over the year.

° = semi-extensive, goats are kept in the fold during winter before the kidding period (January to March) and from March to November they are free to graze and browse.

§ = intensive, goats are reared indoor all over the year.

Treatments against helminths were performed once annually in 73.63% (81/110) of the flocks, but 20.00% (22/110) of farmers declared not regularly treating their goats every year. No differences in frequency of treatments were detected depending on the breeding system and reared breeds. Annual treatments usually occurred in autumn or winter (November to January), depending on the farm and particularly on the dry status of does. A few flocks received an additional treatment in summer when necessary, and a low percentage of farms (2.72%) regularly administered two treatments. The choice of anthelmintic varied among the farms but was dominated by BZs (87.27%); only 2.73% (3/110) of farmers treated their flocks with MLs. Anthelmintics were not rotated on any of the farms. In 89.09% (98/110) of the flocks, anthelmintic dose rates were calculated on the basis of the visually estimated body weight of the goat that appeared heaviest. The dose rate for sheep was used to treat the goats in all these flocks. Eleven of the 110 farmers (10%) did not indicate the dose rate used in their flocks. Annual treatments were usually done without any parasitological analyses, which were only performed on particular occasions (Table 4).

**Table 4:** Responses of 110 goat farmers to a questionnaire about practices of gastrointestinal-nematode control in dairy goats

**Factor**	**Percentage (n)**		
Control of gastrointestinal parasites			
Pasture management	0		
Anthelmintic treatment	100%		
Coprological analysis			
Before treatment	0		
Post treatment	0		
Anthelmintic used			
Benzimidazoles and probenzimidazoles	87.27% (96)	Albendazole	25.45% (28)
		Febendazole	25.45% (28)
		Oxfendazole	8.18% (9)
		Netobimin	23.64% (26)
		Febantel	4.55% (5)
Macrocyclic lactones	2.73%(3)	Ivermectin	0.91% (1)
		Moxidectin	0.91% (1)
		Eprinomectin	0.91% (1)
Nd*	10.00% (11)	-	-
Frequency of treatment/year			
0	20.00%		
1	73.63%		
2	2.72%		
Nd	3.63%		
Time of treatment			
Autumn/winter	82%		
Summer	9%		
Autumn/winter + summer	9%		

*not done.

### Composition of gastrointestinal helminthofauna

All necropsied goats were infected by GINs (P = 100%). *T. circumcincta* was the most abundant (mean A = 558.62) and most frequent (P = 73%) abomasal parasite in all studied goats, followed by *H. contortus* (mean A = 69, P = 46%). Other abomasal species belonging to the subfamily Ostertagiinae (*Ostertagia ostertagi*, *Os. leptospicularis*, *Spiculopteragia spiculoptera* morph *spiculoptera*, *Te. pinnata* and *Te. trifurcata*) were also present but with low abundances and prevalences (Table 5). *Tr. colubriformis* and *N. lanceolatus* (mean A = 444.31 and 41.21, respectively) were the dominant species in the small intestine, whereas *Skrjabinema caprae* and *Oesophagostomum venulosum* had the highest epidemiological parameters in the large intestine (Table 5). All sections of the gastrointestinal tract had high mean worm burdens, varying from 1370.08 to 2862.68 nematodes.

**Table 5:** Mean abundance (A), prevalence (P), importance value (I) and location of gastrointestinal nematodes recovered in dairy goats

**Nematode species**	**A (SD°)**	**Min-Max**	**P (%)**	**I**	
				**Values**	**Categories**^**§**^
Abomasum					
*Teladorsagia circumcincta*	558.62 (896.06)	0-3500	73	92.47	D
*Haemonchus contortus*	69 (288.29)	0-1900	46	7.2	D
*Spiculopteragia spiculoptera* morph *spiculoptera*	2.44 (8.23)	0-40	11	0.06	CO-D
*Teladorsagia pinnata*	0.91 (3.54)	0-21	9	0.018	CO-D
*Teladorsagia trifurcata*	0.33 (2.23)	0-15	2	0.016	CO-D
*Trichostrongylus axei*	0.44 (1.44)	0-5	8	0.008	S
*Ostertagia ostertagi*	0.55 (2.19)	0-10	6	0.008	S
*Ostertagia leptospicularis*	0.44 (1.79)	0-10	6	0.006	S
*Spiculopteragia spiculoptera* morph *mathevossiani*	0.33 (1.65)	0-10	4	0.003	S
*Ostertagia lyrata*	0.11 (0.74)	0-5	2	0.0006	S
Small intestine					
*Trichostrongylus colubriformis*	444.31 (1431.91)	0-7200	34	95.99	D
*Nematodirus lanceolatus*	41.21 (185.87)	0-1000	14	3.56	D
*Nematodirus spathiger*	4.65 (24.12)	0-130	7	0.2	CO-D
*Bunostomum trigonocephalum*	1.83 (5.94)	0-30	13	0.16	CO-D
*Trichostrongylus capricola*	0.68 (3.71)	0-20	3	0.014	CO-D
*Nematodirus* sp	1.03 (4.09)	0-20	7	0.04	CO-D
*Nematodirus filicollis*	0.17 (0.93)	0-5	7	0.007	S
*Trichostrongylus vitrinus*	0.17 (0.93)	0-5	3	0.003	S
*Cooperia pectinata*	0.17 (0.93)	0-5	3	0.003	S
*Strongyloides papillosus*	0.17 (0.93)	0-5	3	0.003	S
Large intestine					
*Skrjabinema caprae*	1059.34 (1321.72)	0-6244	95	89.03	D
*Oesophagostomum venulosum*	50.92 (73.22)	0-275	58	2.61	D
*Chabertia ovina*	2.42 (5.88)	0-20	21	0.04	CO-D
*Trichuris ovis*	4.18 (20.16)	0-123	11	0.039	CO-D
Total adult nematodes					
Abomasum	1370.08 (1999.53)	0-8165	80		
Small intestine	1870.17 (4146.71)	0-19580	78		
Large intestine	2862.68 (3409.54)	0-16337	97		

°SD = standard deviation.

^§^Based on importance values helminth species were classified into categories; Dominant (D), species strongly characteristic of the community (I > 1.0), Codominant (CO-D) species contributing significantly to the community (0.01 < I < 1.0), Subordinate species occurring infrequently, they do not contribute significantly to the community (0 < I < 0.01).

## Discussion

The questionnaire indicated that the anthelmintic classes BZ and ML were used against GINs in Northern Italy and the FECRTs performed in accordance with WAAVP guidelines demonstrated that AR involved all two anthelmintic classes, with an overall prevalence of 33.33% (5/15 flocks selected for AR testing). Resistance and suspected resistance was found in 40% (4/10) of the flocks selected for AR testing that were treated with BZs, while 20% (1/5) of the flocks treated with MLs had resistant GINs. The absence of L3 in the post-treatment coproculture from the flock with suspected resistance to OFZ, however, did not permit an accurate assessment of sensitivity to this drug. Excluding this flock would lower the percentage of flocks presenting AR to BZs to 30% (3/10) and the overall prevalence of AR resistance to 26.67% (4/15).

Very little data is available on AR on Italian goat farms. Resistance to BZs in *Tr. colubriformis* was recently demonstrated in southern Italy [24]. A generic resistance to BZs was reported by Genchi et al. [8], and AR has been reported in Italian sheep [8,25,26]. The presence of AR in goats throughout Europe is extremely variable, probably because several factors affect the development of resistance in GINs. Chartier et al. [27] found that AR to BZs was present in 100% (15/15) of tested flocks in western France, and a recent Norwegian survey detected AR in 7.69% (1/13) of tested flocks, suggesting that AR in Norway was only beginning [28]. AR has been detected at low frequencies in other European countries such as Sweden [29], Germany [30] and Slovakia [31]. BZs are the class of anthelmintic involved in most cases of AR [27,28,32].

AR to BZs in Northern Italy also appears to be more widespread than AR to MLs; this difference is probably due to the long-term use of BZs and the lack of rotation with other drugs on dairy-goat farms in this region. The isolation of L3 from post-treatment coprocultures from flocks with resistance to BZs indicated that only the *Teladorsagia/Trichostrongylus* group was resistant to this anthelmintic class. Reports of AR to ML on European goat farms are rare. AR in *Teladorsagia*, *Trichostrongylus* and *Haemonchus* has been reported in Denmark, Switzerland, Germany and the UK [30,33,34]. The L3 isolated from the T2 coproculture of flock 15 of our study, which were classed as resistant to EPM, were *Teladorsagia/Trichostrongylus* and *Haemonchus.* The T1 and T2 coprocultures from this flock showed that AR probably involved primarily *Teladorsagia/Trichostrongylus. Haemonchus* L3 were the most abundant (39%) in T1 coprocultures, followed by *Teladorsagia/Trichostrongylus* L3 (32%), while the proportion was inverted in T2 coprocultures (75% *Teladorsagia/Trichostrongylus* L3 and 25% *Haemonchus* L3). Nevertheless, AR to EPM in flock 15 appeared to involve both *Teladorsagia/Trichostrongylus* and *Haemonchus*. Even though AR to EPM in goats had already been described in Switzerland and Germany, the results for this flock were quite unexpected. EPM is only registered for use in cattle in Italy, and the questionnaire indicated that its extra-label use occurred in only 0.91% (1/110) of Northern Italy farms. Flock 15 had never been treated with EPM, but the goats had been dewormed once a year for four years with IVM at the dose rate indicated for sheep. This practice in flock 15 may have caused the appearance of AR to the subclass of avermectins or to the entire ML anthelmintic class, despite the low frequency of treatment. The development of AR to the entire ML anthelmintic class (so also to MOX) due to the repeated use of ivermectin is reasonably still uncommon in goats; a treatment with MOX in goats that failed against IVM-resistant *Ostertagia* (*Teladorsagia*) species was described by Leathwick [35] in New Zealand, and the GINs of a goat flock described by Scheuerle et al. [30] were resistant to EPM but still sensitive to MOX. The efficacy of MOX against GINs should be evaluated in flock 15, and generally on goat farms with verified AR to avermectins, because resistance to milbemycins differs from that to avermectins [36]. MOX, if effective, could be used to prevent the development of AR to milbemycins.

The questionnaire submitted to goat farmers of Northern Italy indicated that the annual number of treatments is very low respect to other countries [4,37]. In fact, the farmer answers showed that treatments against GINs were performed once annually in 73.63% of the flocks and less than once annually in 20.00% of the flocks. The low frequencies of treatments in Lombardy probably did not prevent the appearance of AR in GINs, as also suggested by Chartier et al. [38]. Moreover, the questionnaire revealed that errors in dose rates were common. First of all, the majority of farmers (89.81%) declared to treat their goats with a dose rates for sheep and in all farms visual weight estimate was used. As a consequence, underdosing could result and selection of anthelminthic resistant worms was made easy. According to several authors, this event occurs more frequently in goats drenched with BZs as the bioavailability of the drugs is low than in sheep [4,39,40]. In addition, BZs, resulting in this study implicated in AR, were the most used drugs in Northern Italy and the alternation of anthelminthic families was not performed in any farms. Otherwise, very few drugs against GINs are registered in Italy for goats (6) at the same dose rate for sheep and they are not allowed in lactating goats (2 MLs, 1 levamisole/oxyclozanide) or a withdrawal time (min 3-max 9 days) is required (1 BZs, 1 proBZs, 1 morantel). It follows that the alternation of anthelminthic families to prevent AR as suggested is very difficult in Northern Italy [39]. Finally, the questionnaire survey showed poor drench practices widespread among goat farmers that probably generated the AR detected in GINs. However, it should be further considered that the control practice against GINs in goat flocks adopted by farmers in Northern Italy are not effective enough to improve the productivity in milking goats [39]. In fact, in this area the treatments are usually done in autumn-early winter; at this time treatments are less useful to prevent both the development of the disease and the contamination of pasture by nematode eggs being previously demonstrated that highest fecal egg counts in goats from Northern Italy occurred mainly in summer [6].

The survey on the composition of gastrointestinal helminthofaunas by the necroscopy of goats showed that *Te. circumcincta*, *H. contortus*, *Tr. colubriformis*, *Sk. caprae* and *Oesophagostomum venulosum*. were the most abundant and prevalent species of the gastrointestinal tract. Most of the species were previously reported in naturally infected goats in Northern Italy but *N. lanceolatus* is described for the first time in Italian goats [41,42]. According to Rossi [19], *N. lanceolatus*, placed in synonymy with *N. oiratianus* by Samson [43], showed distinctive features of spicul tips, bursal rays and lateral lobes of the caudal bursa. Some of the recorded helminths can also infect wild ruminants. For example, *Sp. spiculoptera* morph *spiculoptera*, *Sp. spiculoptera* morph *mathevossiani* and *Os. leptospicularis* are common parasites in *Cervus elaphus* and *Capreolus capreolus*[44], and *Te. circumcincta* is one of the most dominant species in the Alpine chamois, *Rupicapra rupicapra*[41,44]. The host distributions of these parasites suggest possible interactions between goats and wild ruminants. Similar interactions probably also occur in domestic species: goats and sheep share numerous gastrointestinal parasites (e.g. *H. contortus*), and the presence of *Os. ostertagi* in goats is probably due to its presence in cattle in the same pasture zones. Further, the analysis of helminthic community structure shows that these species, particularly *Sp. spiculoptera* morph *spiculoptera*, give an important contribution to composition of the community even if they have been classified into the categories of codominant or subordinate species by the importance values. In addition, the detection of a few species typical of cervids emphasizes that the goats may incur other parasitic risks resulting from interaction with these hosts on pastures [45]. Overall, a high number of helminth species is found than previous studies [46]. In this respect, it should be noted that the helminthofauna of a few goats from semi-extensive farms sampled in this survey shows a scarce number of helminth species and appears dominated by *Te. circumcincta*. It may result from the few ecological niches offered by that environment to parasites life cycle rather the frequent use of anthelminthics [33,47]. By quantitative analysis, *Te. circumcincta* and *Tr. colubriformis* showed both high prevalence and worm burden in agreement especially with data of Chartier and Reche [46] but disagree with those of Domke et al. [48] that in goats from Norway found very low prevalence and abundance of *Tr. colubriformis*. As suggested by the authors, it may depend on the scarce ability to overwintering on pastures by this parasite. Further, *H. contortus* showed higher prevalence rates than has been previously found in goats from the same area or from different areas with temperate climate [41,46,49]. This parasite is especially frequent in dry areas where higher worm burden are also recorded; however, *H. contortus* has a wide distribution being also found in regions characterized by a very severe climate due its ability to survive inside the hosts [42,49-51]. At last, the presence of high worm burden of *Sk. caprae* in the large intestine is remarkable; this parasite is generally considered with a very low pathogenicity but it can cause in dairy goats restlessness, itching and lesions in the perianal region [52]. In addition, all sector of gastrointestinal tract showed high worm burdens similar to those recorded in previous surveys as in goats from dry areas of Central Spain or Central and northern Greece [40,49]. These finding coupled with the high prevalence values ranging from 80% to 97% of goats with abomasal and intestinal infection respectively demonstrate that the GINs influence is very important in the surveyed area of Northern Italy including a few pastures with alpine features where despite the climatic conditions could be hard the GINs life cycle don’t seem undergo any interruption. The present study provides sufficient data to make a picture of GINs infection in goat farms in Northern Italy by qualitative and quantitative point of view and based on these finding the risk posed by GINs infection on dairy goats raised in Northern Italy seems quite high.

## Conclusions

The established presence of AR in 30% of the tested flocks in Northern Italy suggests that strategies to prevent the development of AR should be widely adopted in this region. The answers to questionnaire show that farmers and practitioners should be firstly educated about the importance of using the correct dose rates in goats and about the rotation of different anthelmintic classes, because AR can develop even at low frequencies of treatment. The management of resistance on the farms presenting AR should include the use of targeted selective treatments to sustain susceptible GINs population in refugia [53]. The questionnaire survey further provide some information on the practices adopted to control GINs in dairy goats that could be useful to improve the treatments against GINs in Northern Italy. However, the results obtained during the survey showed the presence of highly pathogenic GINs, high worm burden in all sector of gastrointestinal tract and high prevalence values. These data also coupled with a few published reports demonstrating some changes on abundance, seasonality and spatial spread of GINs in the last years probably related to climate change [54] suggested that improved diagnosis and active surveillance of GINs infection should be wanted to allow a suitable control of GINs and improve host production. Furthermore, the knowledge of the composition of gastrointestinal helminthofauna and of the epidemiological parameters is also essential to reach this important objective and control the AR.

## Competing interests

The authors declare that they have no competing interests.

## Authors’ contributions

The study idea was conceived by SAZ and MTM. ADC and MV participated in the design of the study; ADC helped administering the questionnaire. SAZ and AG participated in the acquisition of the laboratory data. SAZ and MTM carried out the statistical analysis. Data interpretation was done by all authors. SAZ, MTM and MV drafted the manuscript. All authors contributed to the critical revision of the manuscript for important intellectual content and have seen and approved the final draft.
